# Alaska Arctic Highways LiDAR dataset for advanced infrastructure modeling

**DOI:** 10.1016/j.dib.2026.113239

**Published:** 2026-09-07

**Authors:** Trevor Greene, Nafiul Nawjis, Muhammad Umair, Kyle Foerster, Naima Kaabouch, Timothy Pasch

**Affiliations:** a Artificial Intelligence Research (AIR) Center, University of North Dakota, Grand Forks, North Dakota 58202, USA; b Communications Department, University of North Dakota, Grand Forks, North Dakota 58202, USA

**Keywords:** Arctic, Alaska highways, Road surface segmentation, Remote sensing, Distributed computing, Permafrost roads, Geospatial analytics, High-density point clouds

## Abstract

The Alaska Arctic Highways LiDAR Dataset (AAHLD) is a curated collection of high-resolution 3D point clouds acquired along approximately 95 km of Arctic roadway corridors in Alaska, including the Dalton, Steese, and Nome-Council highways. Collected between 2022 and 2024 using a combination of ground-vehicle and uncrewed-aircraft LiDAR platforms, the dataset comprises 826 spatial tiles per processing stage, organized into raw, segmentation-reference, and filter-reference products, for a total of 2478 LAZ files and approximately 16.14 GiB. The data were captured with a LiDAR USA Surveyor 32 system, which the manufacturer specifies to support vertical accuracy better than 2 cm and point densities exceeding 300 pts/m^2^ under appropriate acquisition conditions. The release also includes Nome-only CSV damage labels documenting 3906 unique damage instances across potholes, cracks, edge cracks, and washed-out road sections. The dataset is openly available through the University of North Dakota (UND) Commons repository at https://commons.und.edu/data/39/ under a Creative Commons Attribution-NonCommercial 4.0 International license. AAHLD is intended to support research in Arctic infrastructure monitoring, road segmentation, road damage detection, and machine learning model development in cold-region transportation environments.

Specifications Table

**Table:** 

Subject	Artificial Intelligence (AI), machine and deep learning, computer vision, transportation infrastructure.
Specific subject area	LiDAR-based mapping and road-surface analysis of Arctic highways in Alaska.
Type of Data	3D point cloud (LAZ); CSV annotation files; CSV coordinate files; ZIP archives; TXT/XLSX support files.
Data collection	Data were collected between 2022 and 2024 with a LiDAR USA Surveyor 32 [1] mounted on ground vehicles and uncrewed aircraft systems [2,3]. Raw PCAP files were converted to LAS/LAZ in QTM, then partitioned into spatial tiles at 100 m intervals along the longest cardinal axis of each surveyed section; roadway footprints were manually segmented, filtered, and road-damage labels were created for a Nome subset as CSV files.
Data source location	Dalton Highway, Steese Highway, and Nome-Council Highway, Alaska, USA; public release stored in University of North Dakota Scholarly Commons (Nome, Prudhoe Bay, and Yukon directories).
Data accessibility	Repository name: University of North Dakota Scholarly Commons. Data identification number: https://doi.org/10.31356/data039. Direct URL to data: https://commons.und.edu/data/39/. Instructions for accessing these data: open the repository record and download the public release; no special access request is needed.
Related research article	None.

## 1 Value of the Data

• The proposed dataset contains LiDAR scans of almost 95 km of Arctic highways in Alaska.
• It includes multiple levels of data refinement, raw, spatially sliced, manually segmented, and noise-filtered, making it suitable for a wide range of technical applications in infrastructure assessment of 3D vision techniques and AI models focused on road segmentation, surface classification, vegetation detection, and damage identification.
• Includes annotated road damages to enhance practical AI/ML applications.
• Its structure is optimized for distributed computing environments, enabling efficient parallelization for training deep learning and reinforcement learning systems at scale.
• The dataset holds operational value for researchers and infrastructure stakeholders by providing high-resolution baseline roadway conditions for Arctic and cold-region settings. It can support road condition assessment, model development, and comparison with future repeat surveys.
• It is openly archived through the UND Commons data repository at https://commons.und.edu/data/39/, with the repository record serving as the authoritative source for the files and accompanying documentation.

## 2 Background

Maintaining Arctic transportation infrastructure is difficult because of long distances, limited access, extreme seasonal variability, and sensitive permafrost terrain. Roads in Alaska are affected by thawing, flooding, erosion, snow accumulation, meltwater, and vegetation encroachment, which makes frequent field inspection challenging and increases the need for high-resolution geospatial data. This article presents the AAHLD, a high-density collection covering nearly 95 km of the Dalton, Steese, and Nome-Council highway corridors in Alaska. The dataset was collected between 2022 and 2024 using both ground-based and uncrewed-aircraft LiDAR platforms and is organized into raw, segmentation-reference, and filter-reference spatial tiles generated at 100 m intervals along the longest cardinal axis of each surveyed section. It also includes Nome-only road-damage labels for potholes, cracks, edge cracks, and washed-out sections. The release is designed to support roadway condition assessment, surface modeling, damage detection, and machine learning workflows in Arctic and cold-region transportation research.

## 3 Data Description

### 3.1 Geographic and environmental coverage

**Fig. 1:**
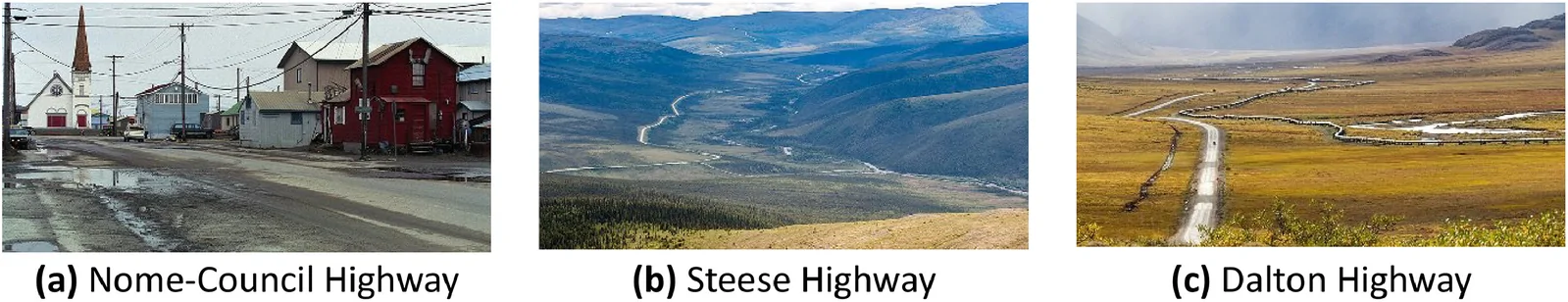
Representative photos of diverse Arctic environments: urban, mountainous, and tundra. Photos courtesy Jerzy Strzelecki, BLM Alaska, T. LaBar via WikiCommons.

The data collection sites span a range of Arctic environments, including flat tundra, mountainous passes, gravel and paved highways, and urban road segments in and around the city of Nome, as shown in Fig. 1.

These roads were selected because of their importance to energy infrastructure, logistical operations, and federal transportation planning. Surveys were conducted from 2022 to 2024, enabling the capture of variation in road conditions that affect noise in the data.

Each surveyed highway section presents unique environmental and topographical features that create challenges for systems relying on automated perception. Because of these diverse ecological and geographical features, the dataset supports evaluation of road-surface modeling across a wide range of Alaskan roadway conditions.

### 3.2 Data volume and repository structure

The dataset described in this article is archived through the UND Commons data repository at https://commons.und.edu/data/39/.

The release contains 826 spatial tiles in each of three processing stages: spatially sliced raw point clouds, segmentation-reference point clouds, and filter-reference point clouds. It therefore contains 2478 LAZ point-cloud files in total. The tiles cover almost 95 km of roadway across the Nome, Prudhoe Bay, and Yukon regions of Alaska. The released archive occupies approximately 16.14 GiB and includes LAZ point clouds, CSV files, ZIP archives, README documentation, a file-to-description workbook, and SHA-256 checksums (Table 1).

**Table 1:** Regional contents of the AAHLD release. Region names reflect the public archive directory structure and correspond to the surveyed Nome-Council, Steese, and Dalton highway collection areas. Point counts are derived from LAZ headers.

Region	Tiles per stage	Raw points	Segmentation points	Filter points	Labels and coverage
Nome	185	966,298,093	297,823,603	297,091,361	152 CSV label files; 3906 unique damage instances
Prudhoe Bay	128	710,440,530	497,271,217	489,728,407	No damage-label product in the release
Yukon	513	1791,542,094	975,267,235	973,033,936	No damage-label product in the release
Total	826	3468,280,717	1770,362,055	1759,853,704	Nome-only damage labels

The collection metadata describes almost 95 km of roadway across the Nome, Steese, and Dalton highway corridors; this total should not be apportioned to individual directory regions without additional verified route data. For this article, point density was calculated as the LAZ header point count divided by the axis-aligned XY bounding-box area of each tile. Release-wide weighted densities were calculated by dividing the summed point count by the summed tile-bounding-box area within each processing stage. This definition provides a reproducible tile-bounding-box density metric and should not be interpreted as a road-surface-density estimate.

Detailed per-tile acquisition metadata, including collection platform, weather, season, vehicle speed, flight altitude, and trajectory logs, are not included in the public release. Vehicle-path coordinate CSV files are included with the Prudhoe Bay and Yukon raw tiles and contain UTM X and Y coordinates, but the supplied documentation does not identify the EPSG code or a vertical datum. This limits precise alignment with external GIS layers, but it does not prevent the main intended uses of the dataset because segmentation, filtering, damage labeling, and ML workflows rely primarily on relative geometry within each tile.

The dataset undergoes refinement through manual road segmentation in QTM [5]. Across all 826 matched raw and segmentation-reference tile pairs in the release, segmentation reduced the total point count from 3468,280,717 to 1770,362,055 points, a weighted reduction of 48.96%. The corresponding compressed LAZ storage volume decreased by 69.07%. These statistics describe point-count and storage reductions rather than roadway-surface point density.

## 4 Experimental Design, Materials and Methods

### 4.1 Survey design and objectives

The AAHLD was developed to generate precise, high-resolution 3D representations of major transportation routes in the Arctic. The effort focused on the Dalton, Steese, and Nome-Council highways, each of which plays a central role in regional coordination, energy infrastructure, and emergency response. These routes were selected not only for their strategic value, but also for the range of environmental conditions they represent, from coastal flood zones and permafrost plains to rugged mountain passes and compact urban corridors. Snow accumulation, meltwater, and seasonal freeze–thaw cycles induce surface deformation and drainage variability, often accelerating road degradation in Arctic [7]. The dataset documents roadway surface conditions captured during discrete survey campaigns and can support infrastructure condition assessment, baseline characterization, and future repeat-survey comparisons. Because freeze–thaw deterioration is a temporal process, the present release should not be interpreted as a continuous time-series dataset for directly measuring seasonal change.

**Fig. 2:**
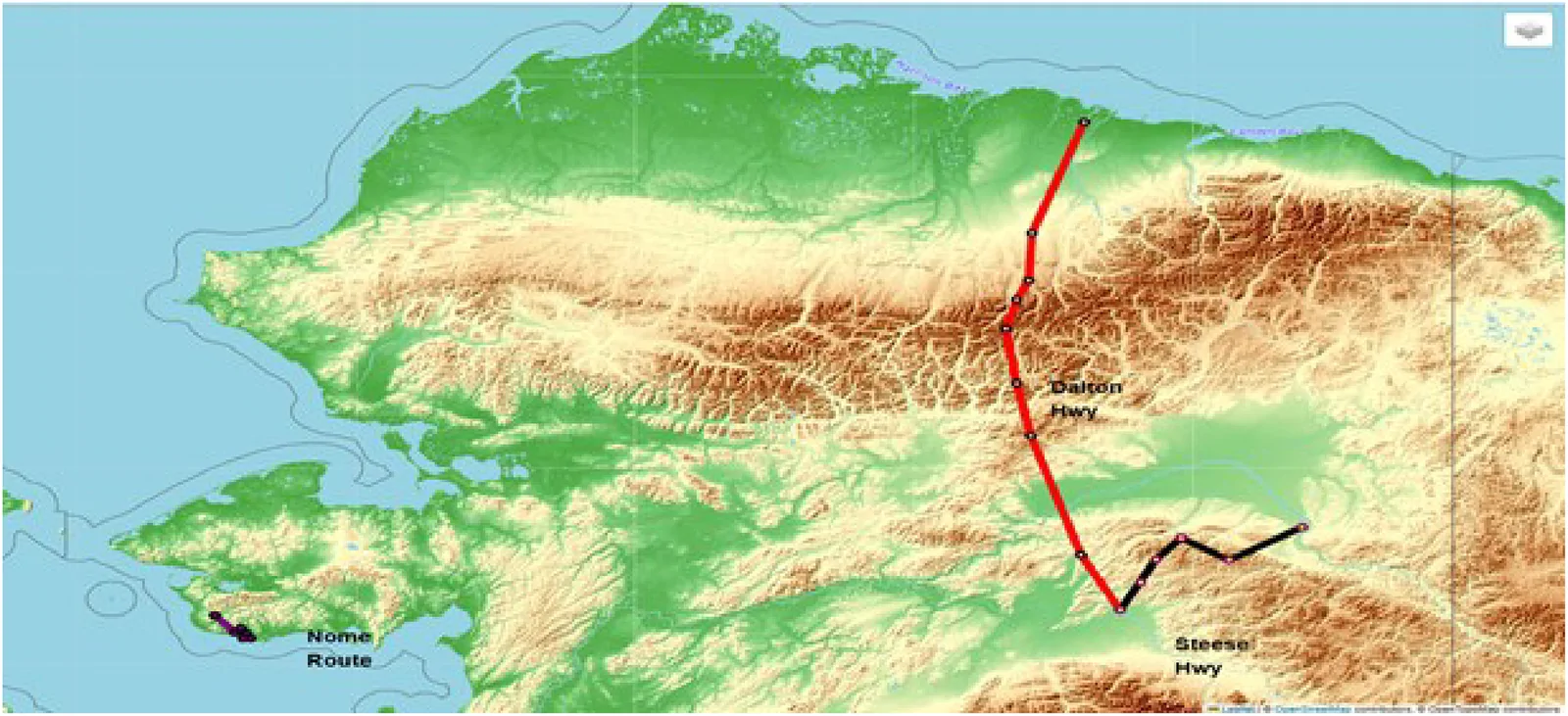
Extent of the released LiDAR survey coverage along the Dalton, Steese, and Nome-Council highway corridors, totaling approximately 95 km of roadway included in the public dataset.

To support strategic route selection and planning, the surveyed segments are illustrated in Fig. 2, which shows the geographic extent of LiDAR coverage across the Nome, Steese, and Dalton highways.

To ensure geographic and environmental diversity, the data collection included urban scans in Nome, high-elevation terrain along the Steese Highway, and long gravel stretches of the Dalton Highway extending into the Prudhoe Bay oil region. Acquisition methods were adjusted to the terrain: UAS (Unmanned Aerial System) were deployed for remote or mountainous areas, while ground vehicles carrying the LiDAR sensor were used where road access allowed. Each mission captured the road surface and adjacent features using acquisition settings intended to support high-density scanning. The 300+ pts/m${}^{2}$ and <2 cm values are acquisition-system specifications. Using LAZ header point counts and each tile’s axis-aligned XY bounding-box area, the release has weighted tile-bounding-box densities of 156.12 pts/m${}^{2}$ for raw tiles, 270.31 pts/m${}^{2}$ for segmentation-reference tiles, and 272.18 pts/m${}^{2}$ for filter-reference tiles. These density values characterize the spatial extent of the released tiles rather than the density of points on the roadway surface itself.

**Fig. 3:**
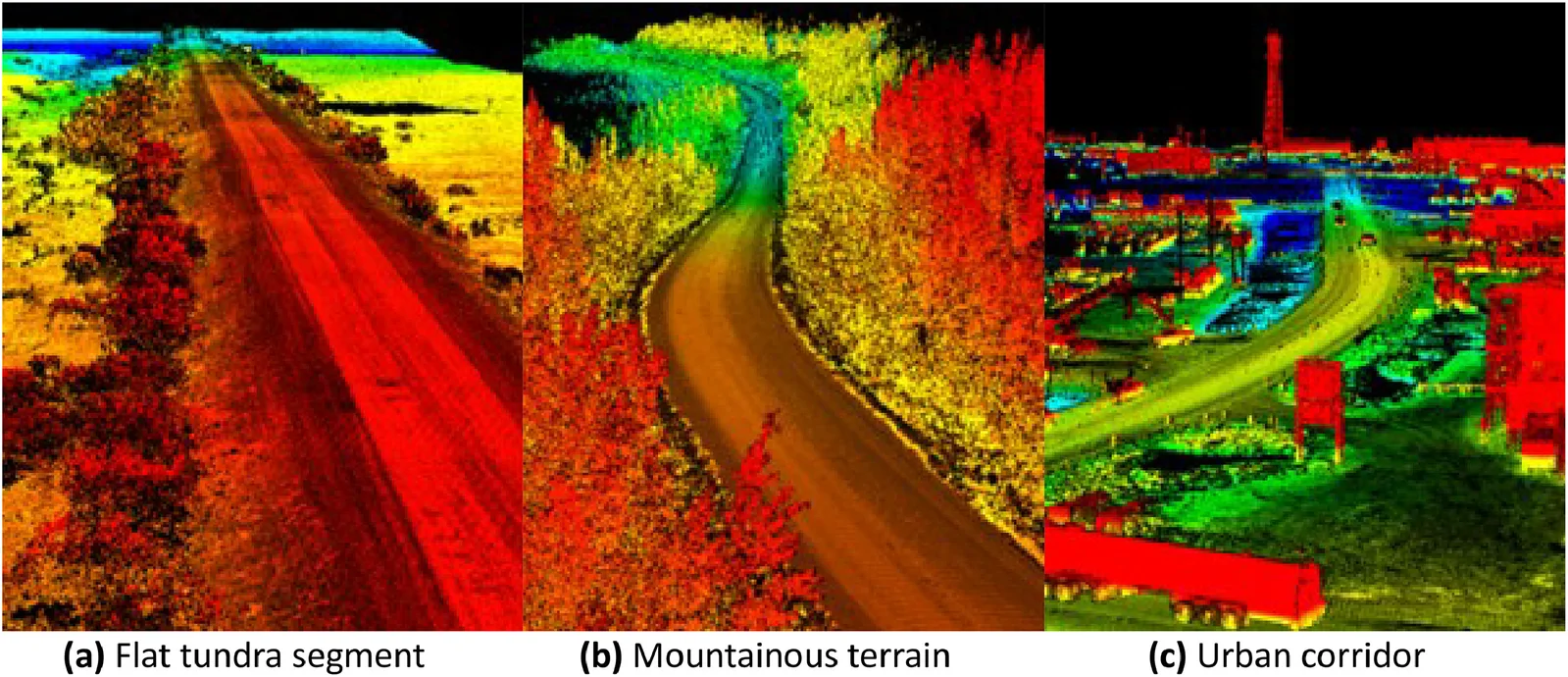
Segmented LiDAR point clouds illustrating different roadway contexts.

To illustrate the variation in terrain and environmental context, three segmented LiDAR images are shown in Fig. 3. Fig. 3a captures a flat tundra section near Nome, where smooth elevation profiles and minimal roadside clutter yield a clean scan. Fig. 3b presents a mountainous section of the Steese Highway, featuring elevated slopes, denser vegetation, and sharper topographical transitions. Fig. 3c highlights an urban corridor in Nome, with clearly segmented roadway boundaries and visible roadside infrastructure such as signage and utility poles. Together, these figures reflect the dataset’s capacity to support modeling across diverse Arctic conditions.

### 4.2 LiDAR sensor and platform specification

Capturing reliable 3D data across Arctic Alaska’s diverse and often inaccessible landscapes required a careful blend of aerial and ground-based LiDAR platforms. Given the range of surface types, including snow-covered tundra, mountainous grades, gravel roads, urban corridors, and data collection strategies had to be both adaptable and technically rigorous. The fieldwork was conducted across varied terrain categories using two primary acquisition platforms: a UAS and a vehicle-mounted LiDAR unit. The available release metadata does not record the specific seasons in which each campaign was conducted.

For accessible routes, such as the Dalton and Steese Highways, ground surveys were performed using a vehicle mounted with a Surveyor 32 LiDAR system, which enabled high-fidelity scanning. For capturing urban segments such as Nome’s Front Street and remote roads that were difficult to survey from the ground, such as Dexter Bypass an uncrewed aircraft was utilized.

All point cloud data in this dataset were acquired using the LiDAR USA Surveyor 32, a multi-channel laser scanning system whose detailed specifications are summarized in Table 2 [1,8]. The manufacturer-specified wavelength, point rate, and density values are device-level specifications and were not independently measured from the released LAZ files.

**Table 2:** Manufacturer-reported technical specifications of the LiDAR USA Surveyor 32 system; these values were not independently validated from the released LAZ files.

Parameter	Value
Laser channels	32
Camera	Dual 24 MP with 25 mm lens
Laser Wavelength	903 nm (near-infrared; Class 1 eye-safe)
Points per second	1280,000
Point density	300+ pts/m^2^
Accuracy at 40 m AGL	<2 cm
GNSS receiver	L1/L2 dual-frequency

The system was paired with a dual-frequency GNSS receiver (L1/L2) and a tightly coupled IMU to ensure robust trajectory correction, critical for minimizing drift and error across long distances and during abrupt terrain shifts.

### 4.3 Aerial surveys

**Fig. 4:**
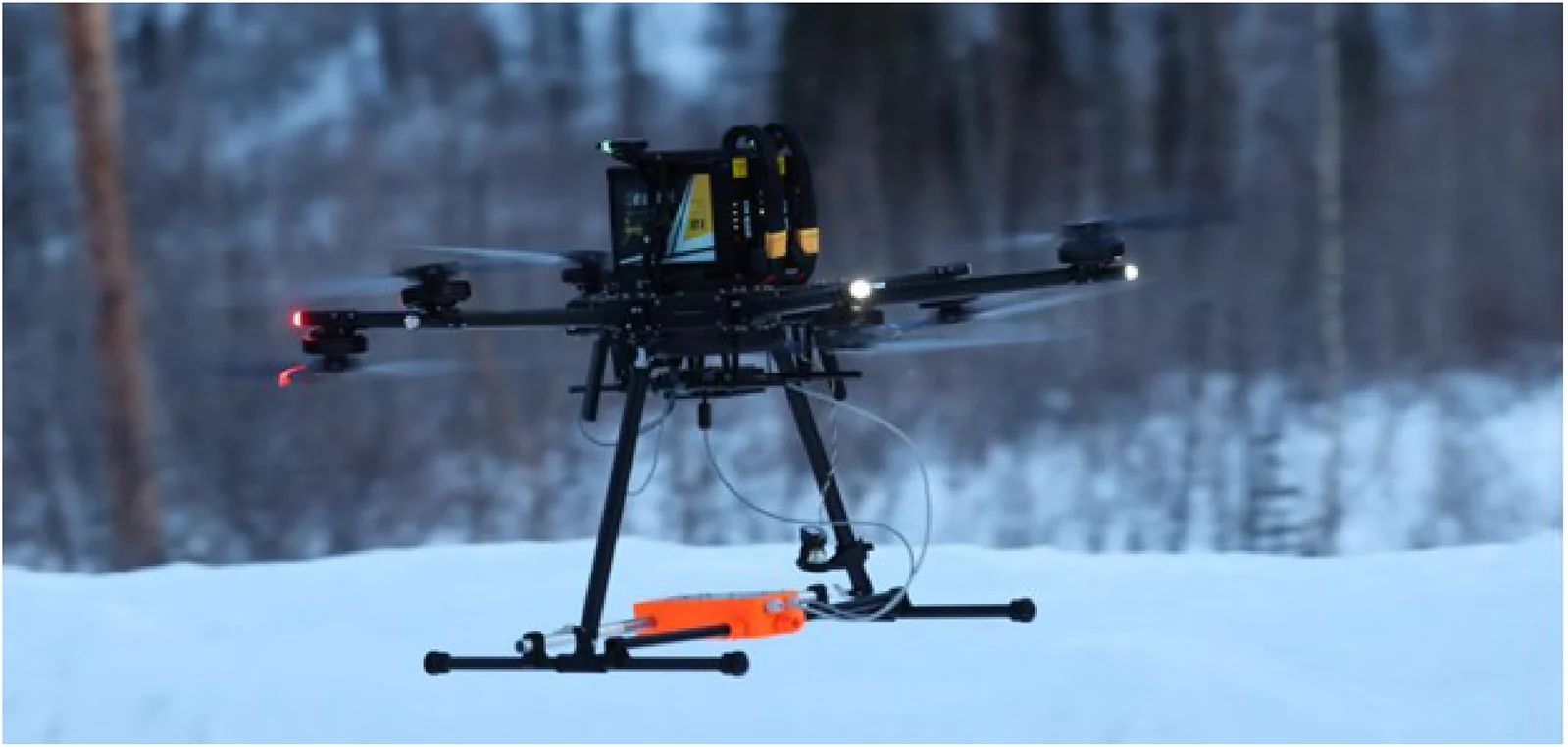
Blue UAS certified aerial platform equipped with a Zond Aero LF variable-frequency ground-penetrating radar (GPR) sensor. The same class of platform was used to carry the LiDAR USA Surveyor 32 system for LiDAR data collection in this release.

In urban and topographically difficult areas, Blue UAS certified Inspired Flight IF1200A and Parrot Anafi USA uncrewed aircraft systems were used as the aerial platforms. These platforms carried the Surveyor 32 LiDAR system for LiDAR data acquisition and were flown over sectioned roadways in Nome, as well as mountain passes that ground vehicles couldn’t safely traverse. The sensor visible in Fig. 4 is a Zond Aero LF variable-frequency ground-penetrating radar (GPR) unit; the same class of aerial platform was also used to carry the LiDAR USA Surveyor 32 system for the LiDAR data in this release. Their stable flight dynamics and payload capacity enabled precise capture over variable terrain without sacrificing resolution.

Drone flights were programmed to maintain consistent speed and altitude, ensuring spatial uniformity and high overlap in scan coverage. Locations such as Snake River Road and Nome-Council Road were captured entirely using UAS operations, particularly during periods when ground traffic or storm debris made vehicle access impractical.

### 4.4 Ground-based survey

**Fig. 5:**
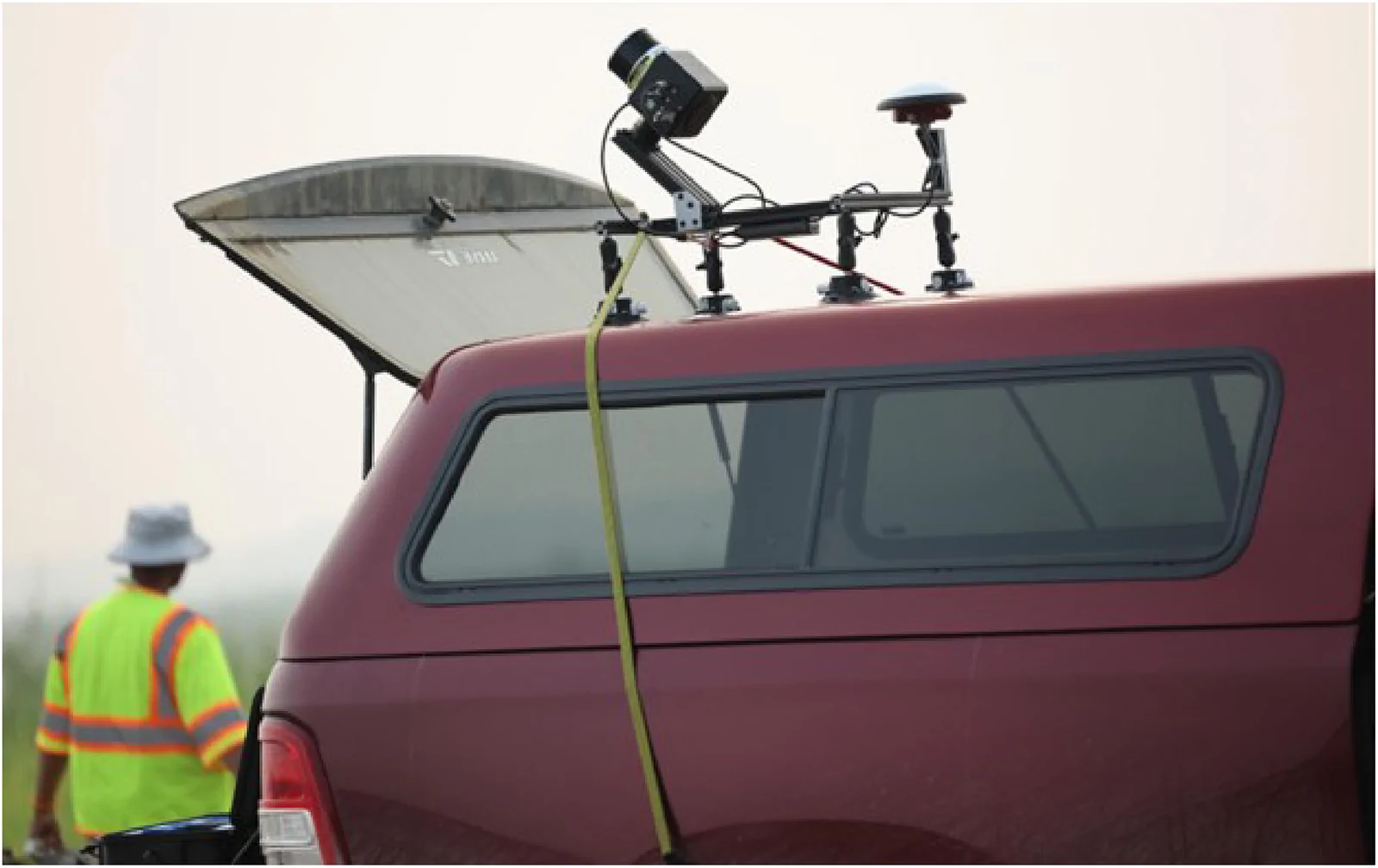
Surveyor 32 LiDAR mounted on a sport utility vehicle (SUV) for mobile data acquisition along Arctic highways.

For extended routes such as the Dalton and Steese highways, the Surveyor 32, shown in Fig. 5, was mounted on an SUV using a secure bracket system. The vehicle operated at approximately 65 km/h while syncing with a GNSS base station nearby. This configuration made it possible to capture long, uninterrupted point cloud strips with minimal vibration interference and solid GPS signal lock.

### 4.5 Dataset generation pipeline

To support advanced applications in computer vision and geospatial analysis tasks, the dataset was processed to balance data fidelity with computational efficiency. The pipeline enables researchers to select subsets of data based on the level of abstraction required for specific tasks, ranging from low-level modeling to AI model training and validation.

### 4.6 Raw data acquisition

**Fig. 6:**
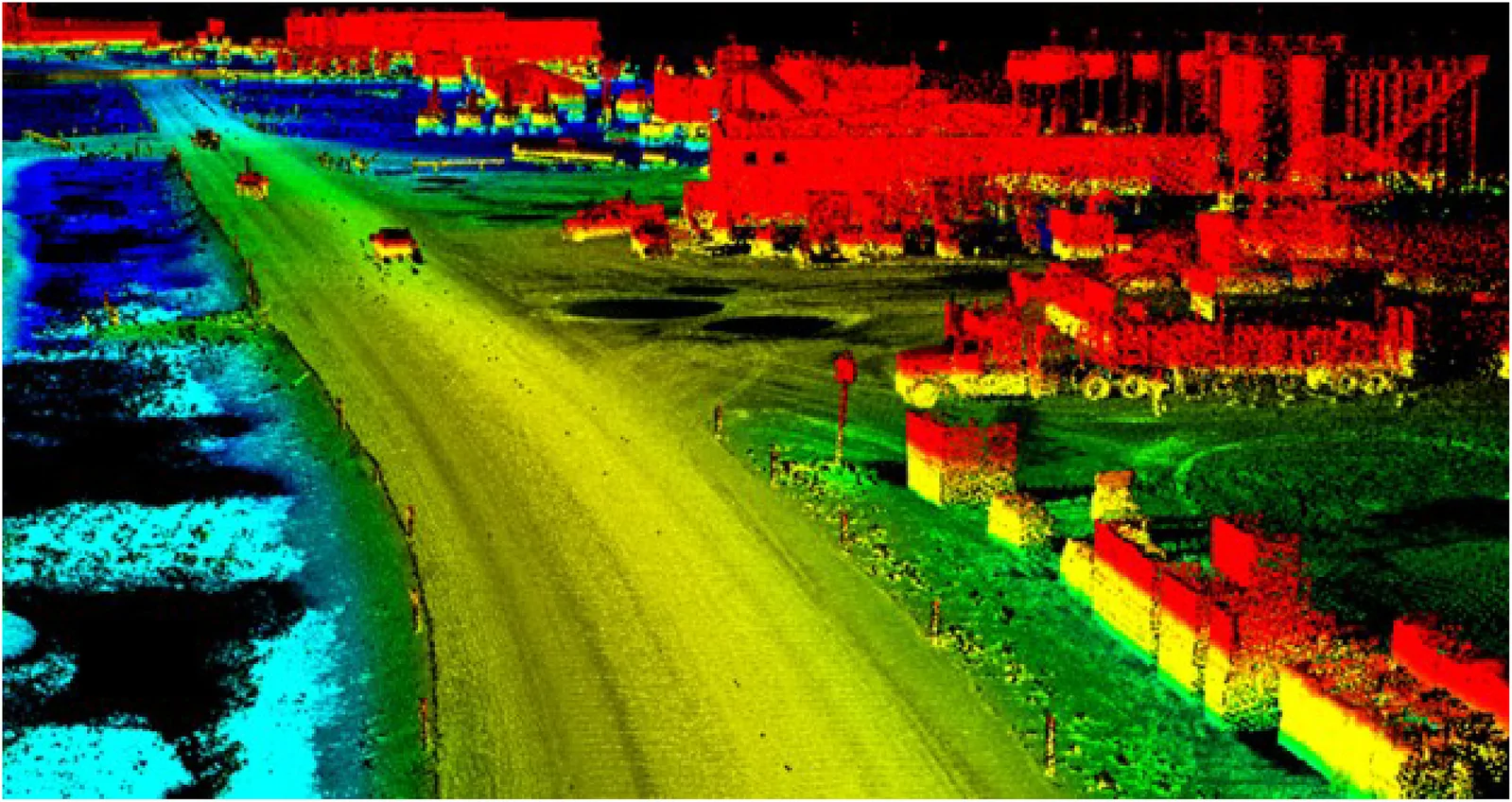
Example of raw 3D LiDAR point cloud data collected at Prudhoe Bay, including roadway surfaces and surrounding environmental clutter prior to processing.

The raw scans capture a high level of detail but also contain significant extraneous elements, such as buildings, vegetation, trees, vehicles, and even dust emitted by the moving vehicle. This visual noise complicates downstream processing, particularly given the substantial file sizes. Fig. 6 illustrates a representative example of unprocessed data, including the various sources of clutter.

During acquisition, raw sensor data were recorded in PCAP format and later converted to LAS and LAZ point clouds using QTM. The public release described in this article contains the processed LAZ products; the original PCAP files are not included in that release. The released LAZ files retain the point-cloud geometry and attributes required for the documented processing products, but detailed acquisition and trajectory metadata are not available on a per-tile basis.

The converted files work with many standard tools used in mapping and analysis, such as Point Data Abstraction Library (PDAL) [6], Open3D [10], Quantum Geographic Information System (QGIS), and ArcGIS. This makes it easier to use the data in workflows for ML, mapping, or infrastructure analysis, whether in research or field settings.

### 4.7 Spatial slicing

**Fig. 7:**
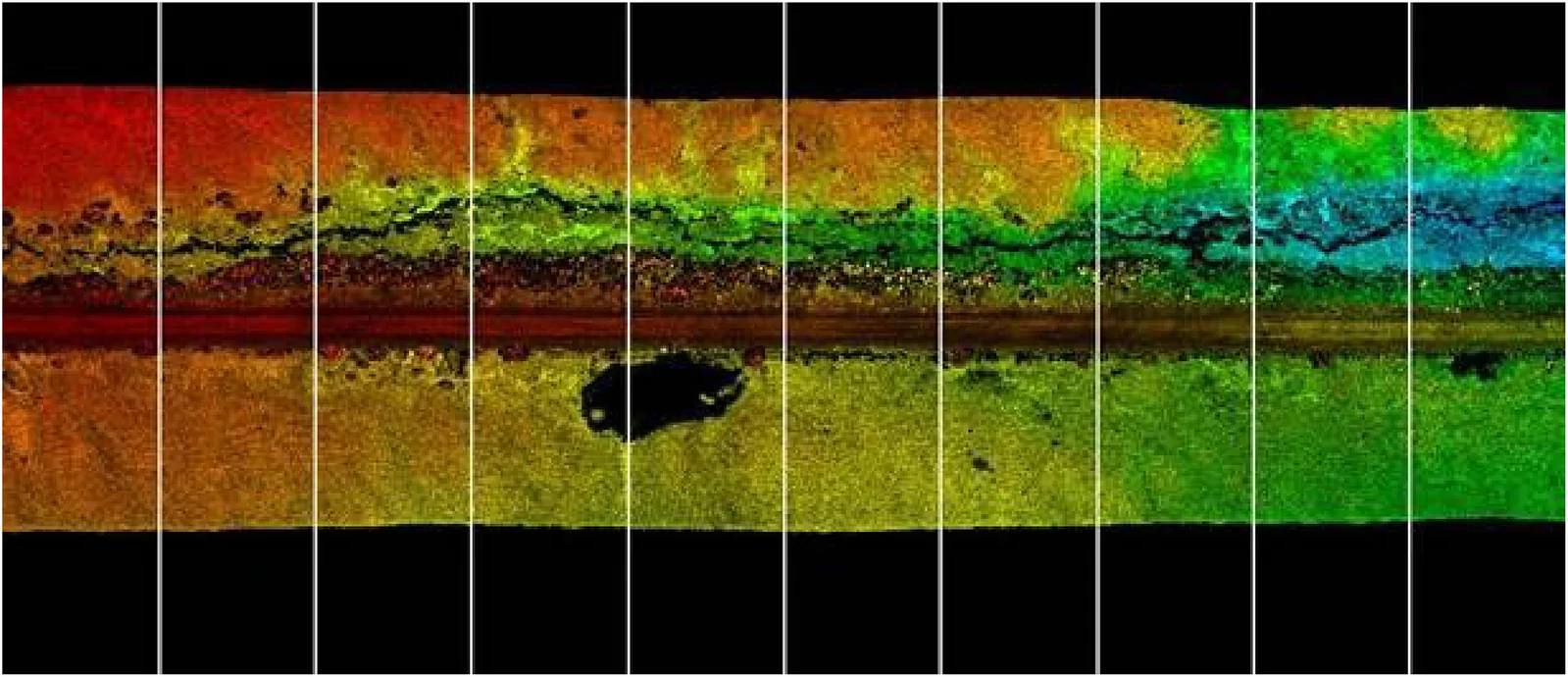
Spatial slicing of a raw LiDAR point cloud into spatial tiles generated at 100 m intervals along the longest cardinal axis of the surveyed section for distributed processing.

The raw LiDAR point clouds were partitioned into smaller spatial tiles at 100 m intervals along the longest cardinal axis of each surveyed section. This made it easier to visualize and process the data, especially for AI tasks, without losing important geospatial or elevation details. The files can be loaded independently, enabling flexible use in both standard desktop environments and distributed parallel computing systems. An illustration of this slicing process is provided in Fig. 7, which shows the division of a point cloud section along the Dalton Highway.

The slicing was performed using a Python-based algorithm developed by the research team. The slicing operation preserves the point coordinates contained in the source point cloud while partitioning each surveyed section into smaller spatial tiles. Each file retains full resolution and includes all original features, such as intact and damaged road surfaces, roadside infrastructure (e.g., poles, curbs, signs), vegetation, buildings, and incidental artifacts such as airborne dust or passing vehicles.

The 2478 released LAZ files contain 6998,496,476 points in total. Using LAZ header point counts and each tile’s axis-aligned XY bounding-box area, the weighted tile-bounding-box density is 156.12 pts/m${}^{2}$ for raw tiles, 270.31 pts/m${}^{2}$ for segmentation-reference tiles, and 272.18 pts/m${}^{2}$ for filter-reference tiles. These density values characterize the spatial extent of the released tiles and are not road-surface-density estimates.

### 4.8 Road segmentation

The segmentation-reference product defines the horizontal roadway extent for mask-style road segmentation. Road boundaries were manually placed at the pavement edge or, for gravel roads, at the point where the road surface begins to curve toward or meet adjacent vegetation. Steep shoulders, drainage ditches, embankments, guardrails, and other roadside areas were excluded. Connected side roads were retained when they formed a complete road surface.

**Fig. 8:**
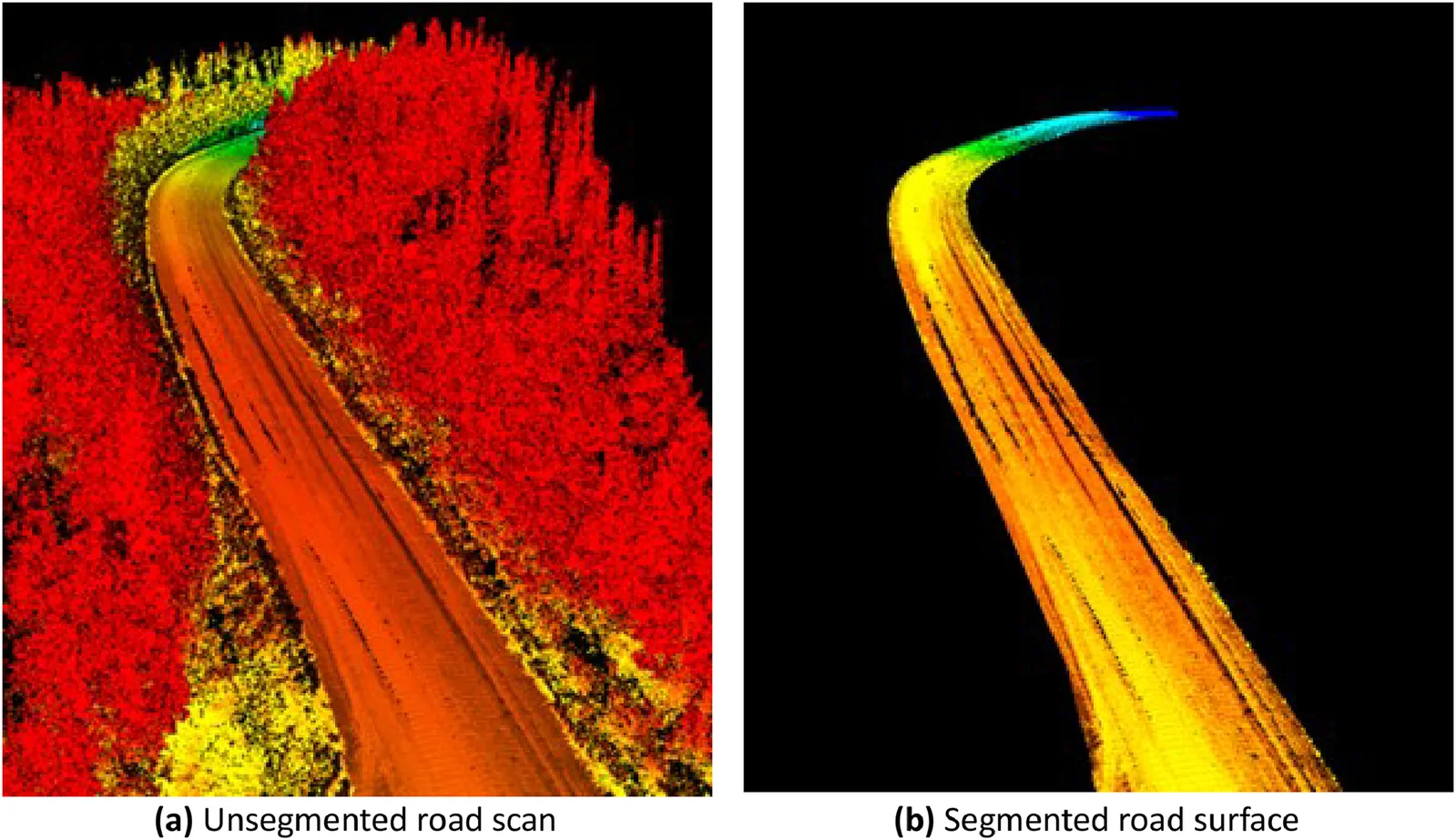
Roadway point clouds before and after manual segmentation using QTM.

The segmentation-reference files intentionally retain non-road objects located above the selected roadway footprint, including vehicles, signs, vegetation, and dust. They should therefore be interpreted as spatial road masks rather than cleaned road-surface point clouds. This design supports evaluation of mask-style segmentation methods, for which an additional filtering step is required to remove objects above the roadway. The filter-reference files provide the corresponding road-surface-only target for evaluating non-mask segmentation methods (Fig. 8).

Point cloud before segmentation and the corresponding segmentation-reference roadway footprint. Objects above the roadway are intentionally retained in the segmentation-reference product for mask-style segmentation evaluation.

Point-count reductions were calculated from LAZ header point counts for matching raw and segmentation-reference files. The release-wide percentage was computed from the summed point counts across all matched tile pairs, rather than by averaging individual tile percentages. Compressed storage reduction was calculated separately from the corresponding LAZ file sizes.

### 4.9 Noise filtering

The filtering stage was applied after the roadway footprint had been defined. Its purpose was to produce a road-surface-only reference product by removing non-road returns above or beside the roadway, including vehicles, vegetation, signs, roadside structures, isolated points, and most airborne dust. Filtering was performed through manual visual editing in Quick Terrain Modeler (QTM), with PDAL used where appropriate in the processing workflow.

Some raised dust or track-like returns were retained when they formed a surface connected to the roadway and could not be removed confidently without also deleting valid road points. The filter-reference products therefore represent substantially cleaned roadway surfaces, but they are not guaranteed to be free of every residual artifact.

The processing workflow relied primarily on manual visual editing in QTM rather than on a fixed numerical threshold set applicable to every tile. Road-boundary decisions followed the roadway-footprint criteria described above, while filtering decisions were made by visual inspection of non-road objects and potential residual artifacts. PDAL was used where appropriate within the workflow, but a complete fixed PDAL pipeline and per-tile parameter log are not available for the public release.

The outcome of this effort is a substantially cleaner roadway point cloud, where the road geometry is preserved with high precision while acknowledging that isolated residual artifacts may remain. The dataset produced through this stage is particularly well-suited for ML/DL applications such as road damage detection, surface classification, and elevation profiling. Its quality and consistency make it suitable for use as reference data in model training and validation. Because isolated residual non-ground artifacts may remain after processing, users whose applications require completely artifact-free roadway surfaces should inspect the relevant slices and apply additional task-specific filtering if necessary.

**Fig. 9:**
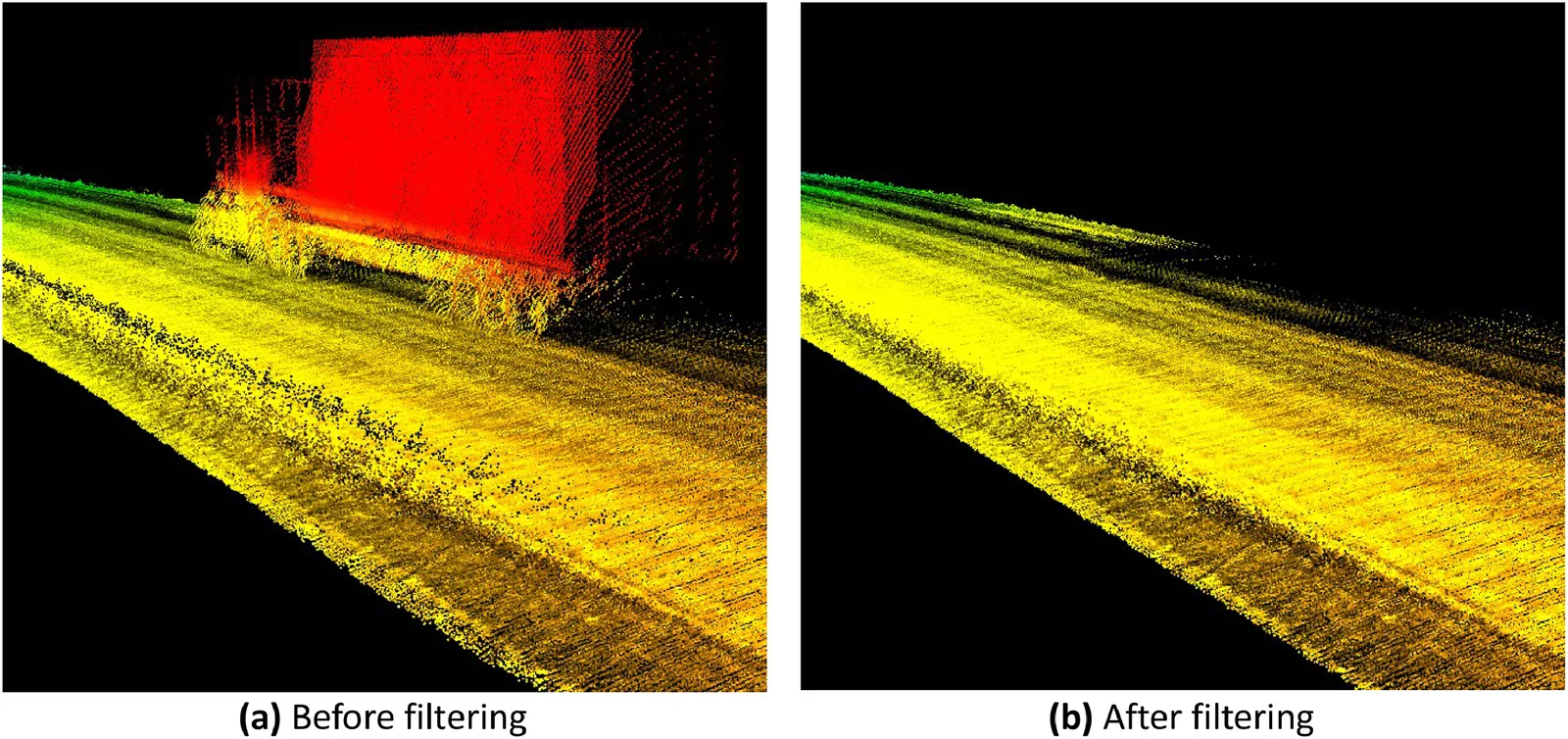
Noise filtering results: removal of artifacts while preserving road geometry.

As shown in Fig. 9, the filtered road cross-section exhibits strong spatial coherence. The filtering operation removes most residual non-road returns while preserving a road-surface-focused reference product. This reference-quality dataset is intended to support AI-based infrastructure analysis, including road damage detection, surface classification, and elevation profiling.

### 4.10 Road damage labeling

The final step in enhancing the AAHLD was the systematic annotation of road damage. Due to the severe and variable weather conditions in the region, the roads captured in the dataset are highly susceptible to multiple forms of deterioration. By introducing structured damage labeling, the dataset enables the development and validation of reliable, real-time detection systems.

**Fig. 10:**
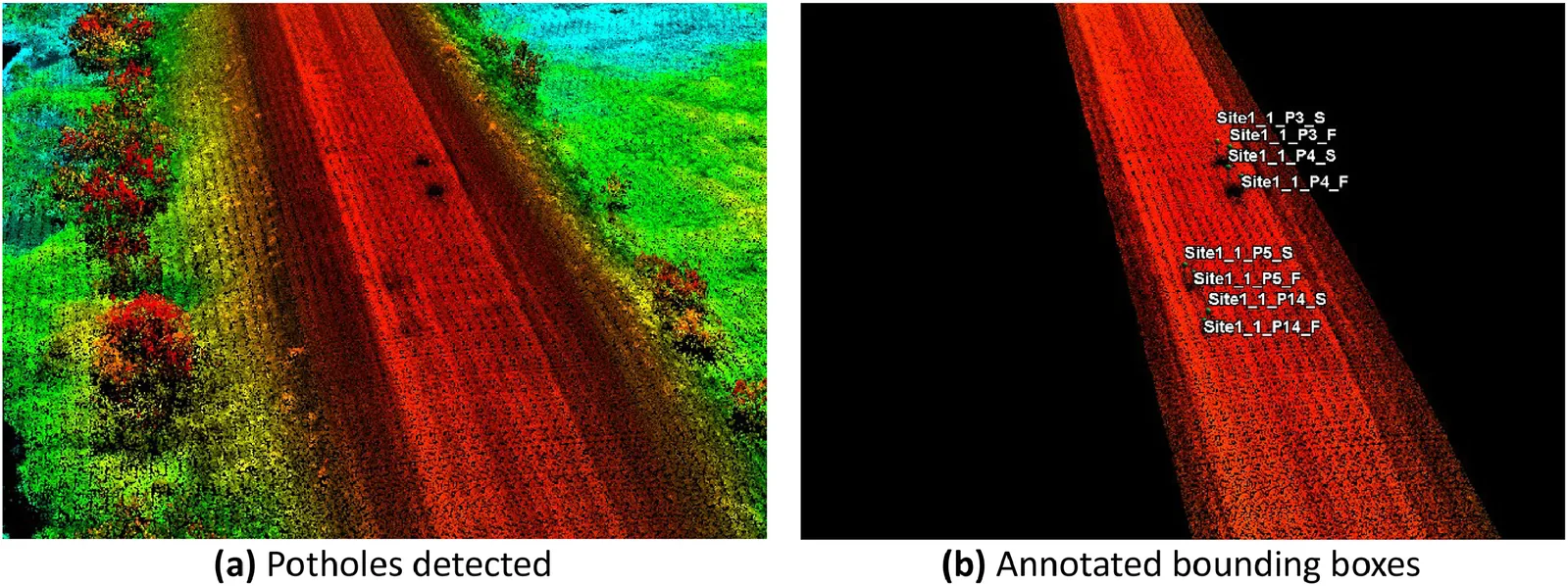
Examples of damaged road surfaces and corresponding annotations.

The annotation process was performed using QTM software and following a two-stage workflow. First, three-dimensional road analysis based on elevation differences was used to identify damaged roadway areas, as shown in Fig. 10a, where multiple large potholes are visible in the LiDAR data.

The released damage labels were reviewed by the original annotator during the annotation process but were not independently assessed by a second annotator. Consequently, no inter-annotator agreement statistic is available. The labels should be treated as single-annotator reference annotations rather than independently validated ground truth.

Second, the start and end points of each damaged area were defined using a Cartesian coordinate system, which allowed bounding boxes to be drawn around the affected portions with each instance of damage labeled by its site name and type of damage as shown in Fig. 10b.

The released labels are provided as CSV files containing a damage identifier, damage type, and three-dimensional bounding-box coordinates. QT Marker files and annotation snapshots are not included in the public release.

The labeled damages were grouped into categories, namely potholes, cracks, edge cracks (sedimentation) and severely affected flood zones, completely washed-away road sections. Each annotation was assigned a code corresponding to its damage type, ensuring consistent classification across the dataset as shown in Tables 3 and 4.

**Table 3:** Intended annotation schema for road-damage labeling in the Nome subset.

LEGEND	
**P**	Pothole
**C**	Crack
**E**	Edge Crack (sedimentation)
**Fl**	Flushed or washed-away road section
**S**	Starting points of bounding box
**F**	Finishing point of bounding box
**NL**	No-Label

**Table 4:** Nome damage-label coverage and class balance in the release.

Measure	Count	Notes
Nome CSV label files	152	All files contain at least one annotation row
CSV annotation rows	4064	Includes paired start and finish coordinate records
Unique damage instances	3906	Instance-level count after pairing endpoint records
Potholes	3809	Unique instances
Cracks	28	Unique instances
Edge cracks	7	Unique instances
Washed-out road sections	61	Unique instances
Unknown damage type	1	Reported separately; not assigned to a damage class
Classified instances	3905	Sum of the four defined damage classes

Annotation schema for road damage classification, including codes for potholes, cracks, edge cracks, and washed-out sections. Annotation counts were calculated from the Damage_Type column across all 152 Nome CSV label files. When a damage identifier contained paired start and finish records, indicated by _S_<n> and _F_<n> suffixes, the pair was counted as one damage instance. All other annotation rows were counted as individual instances.

For example, the label Site3_0_P_S_1,471,597.16,7160,479.4598,54.960 corresponds to Site 3 of the Nome mission, Slice 0 denotes the initial slice. The P indicates that the damage identified was a pothole. The S indicates the coordinate is the starting point of the damage, and the number after the S indicates that it is the first instance of damage in the slice, with coordinates provided in the X, Y, and Z dimensions. All entities follow a comma-separated format (CSV), allowing seamless integration into commonly used AI platforms for training and testing. An illustration of the above file is presented in Table 5.

**Table 5:** Example of a road damage annotation entry, showing site number, slice, damage category, bounding box coordinates, and elevation values in CSV format.

Site3_0_P_S_1,471,597.16,7160,479.4598,54.960							
Site No.	Slice #	Pothole	Start	Number	X	Y	Z
Site3	0	P	S	1	471,597.16	7160,479.46	54.96

## Limitations

The segmentation-reference and filter-reference products have distinct interpretations. Segmentation-reference files define the roadway footprint for mask-style segmentation and intentionally retain objects above that footprint. Filter-reference files remove non-road objects to approximate a road-surface-only output for non-mask segmentation methods. Some raised dust or track-like returns may remain in filter-reference files when they cannot be removed without risking deletion of valid roadway points.

Processing quality control consisted of a rapid visual pass over the generated files and additional inspection of files that produced unexpected results. No independent full-dataset review or formal inter-reviewer agreement analysis was completed. Users should therefore treat both products as reference datasets and perform application-specific inspection or filtering when needed.

The AAHLD was collected during discrete survey campaigns rather than as a continuous monitoring program. Therefore, the current release is best suited for baseline road-condition assessment, surface modeling, damage-label development, and comparison with future repeat surveys. Although the dataset captures road surfaces in Arctic environments affected by freeze–thaw processes, it should not be used by itself to directly quantify seasonal freeze–thaw deterioration unless paired with repeat observations from the same locations.

The AAHLD is released under the Creative Commons Attribution-NonCommercial 4.0 International (CC BY-NC 4.0) license [4]. This dataset is made available exclusively for non-commercial purposes, including academic research, educational use, and public sector infrastructure analysis. Use for commercial development, resale, or redistribution is not authorized under the terms of the current data access agreement.

## Ethics Statement

The LiDAR surveys we conducted exclusively along public roadways, including the Dalton, Steese, and Nome highways, and did not involve access to private property or the collection of personally identifiable information (PII).

No human subjects, animals, or protected ecological zones were involved in the acquisition of the dataset. All aerial surveys were performed in compliance with Federal Aviation Administration (FAA) regulations governing UAS operations in the state of Alaska.

The resulting point cloud data contains only geospatial and elevation information relevant to infrastructure and environmental modeling. Incidental scans of residential or commercial properties were captured solely from within the public right-of-way. Data filtering and segmentation procedures were applied to remove extraneous structures from the final dataset.

The dataset is intended solely for research, educational, and infrastructure planning purposes. Its availability through the UND Commons repository is governed by the license and terms specified in that repository record. No commercial surveillance or targeting applications are supported or authorized.

## Credit Author Statement

**Trevor Greene:** Writing - original draft, Writing - review & editing; **Nafiul Nawjis:** Data Curation, Writing - review & editing; **Muhammad Umair:** Data Curation, Writing - review & editing**; Kyle Foerster:** Data Curation, Writing - review & editing; **Naima Kaabouch:** Supervision, Writing - review & editing, Funding acquisition; and **Timothy Pasch:** Supervision, Project administration, Funding acquisition;

## Data Availability

University of North Dakota Scholarly CommonsLiDAR Datasets for Development and Testing of Preprocessing and Artificial Intelligence Damage Detection of Alaskan Arctic Roads (Original data)

## Appendix: Aerial Platforms and Ground-Penetrating Radar Sensor Details

The aerial data-collection platforms used in this work are the Blue UAS certified Inspired Flight IF1200A and Parrot Anafi USA uncrewed aircraft systems [2,3]. Blue UAS certification is administered through the Defense Innovation Unit Blue UAS program(https://mfe-is.com/blueuas/).

The sensor visible in Fig. 4 is the Zond Aero LF, a variable-frequency ground-penetrating radar (GPR) unit manufactured by SPH Engineering [9]. Product details are available at https://shop.sphengineering.com/products/zond-aero-lf. The LiDAR data described in this release were acquired with the LiDAR USA Surveyor 32 system mounted on the same class of aerial platform.
